# Kif1bp loss in mice leads to defects in the peripheral and central nervous system and perinatal death

**DOI:** 10.1038/s41598-017-16965-3

**Published:** 2017-11-30

**Authors:** Caroline S. Hirst, Lincon A. Stamp, Annette J. Bergner, Marlene M. Hao, Mai X. Tran, Jan M. Morgan, Matthias Dutschmann, Andrew M. Allen, George Paxinos, Teri M. Furlong, Sonja J. McKeown, Heather M. Young

**Affiliations:** 1 Department of Anatomy and Neuroscience, The University of Melbourne, Victoria, 3010 Australia; 2 Florey Institute of Neuroscience and Mental Health, The University of Melbourne, Victoria, 3010 Australia; 3 Department of Physiology, The University of Melbourne, Victoria, 3010 Australia; 4 Neuroscience Research Australia and School of Medical Sciences, The University of New South Wales, 2031 NSW, Australia; 5 Cancer Program, Monash Biomedicine Discovery Institute and Department of Anatomy and Developmental Biology, Monash University, Victoria, 3800 Australia

## Abstract

Goldberg-Shprintzen syndrome is a poorly understood condition characterized by learning difficulties, facial dysmorphism, microcephaly, and Hirschsprung disease. GOSHS is due to recessive mutations in *KIAA1279*, which encodes kinesin family member 1 binding protein (KIF1BP, also known as KBP). We examined the effects of inactivation of *Kif1bp* in mice. Mice lacking Kif1bp died shortly after birth, and exhibited smaller brains, olfactory bulbs and anterior commissures, and defects in the vagal and sympathetic innervation of the gut. *Kif1bp* was found to interact with *Ret* to regulate the development of the vagal innervation of the stomach. Although newborn *Kif1bp*^−/−^ mice had neurons along the entire bowel, the colonization of the gut by neural crest-derived cells was delayed. The data show an essential *in vivo* role for KIF1BP in axon extension from some neurons, and the reduced size of the olfactory bulb also suggests additional roles for KIF1BP. Our mouse model provides a valuable resource to understand GOSHS.

## Introduction

Goldberg-Shprintzen syndrome (GOSHS, OMIM 609460) is a rare human condition characterized by intellectual disability, microcephaly, dysmorphic facial features, peripheral neuropathy as well as Hirschsprung disease in which neurons are absent from the distal bowel^1–4^. GOSHS is associated with recessive mutations in the gene encoding kinesin family member 1-binding protein (*KIF1BP*; also known as *KBP* and *KIAA1279*)^2,4,5^. Studies of human, mouse and zebrafish have shown that *KIF1BP* is expressed in nearly all tissues^5–7^ (www.brain-map.org/). Despite the widespread expression of *KIF1BP*^5^, GOSHS primarily affects the development of neurons and neural crest-derived tissues^4,6^. However, there is a wide range of phenotypic variations between GOSHS patients, and mesenchymal tissues can also be affected, including urogenital structures, limbs, eyes and heart^2–4,8^. Most mutations found in GOSHS patients are thought to cause loss of function of KIF1BP, primarily through nonsense mediated mRNA degradation^2^.

Kinesins (KIF proteins) are a large family of microtubule-based intracellular motors that transport cargo from one part of the cell to another. Cell biological approaches revealed that KIF1BP interacts with a subset of kinesins including KIF1A, KIF1B, KIF1C, KIF3A, KIF13B, KIF14, KIF15 and KIF18A^9–11^. Studies using cell lines and zebrafish mutants suggest the main role of KIF1BP is to regulate axon extension. Depletion of KIF1BP from PC12 cells and the SH-SY5Y neuroblastoma cell line reduced neurite length^2,6^, while overexpression of KIF1BP resulted in increased neurite length in SH-SY5Y cells^2^ but in reduced axon length in cultured hippocampal neurons^9^. *kif1bp* mutant zebrafish exhibit abnormal CNS and peripheral axon outgrowth^7,11^. However, *kif1bp* mutant zebrafish have neurons along the entire length of the bowel and thus do not develop a Hirschsprung disease-like phenotype^7^.

Mutant mouse models have been critical in understanding many developmental diseases. Here we describe the effects of lack of Kif1bp in mice by generating mice in which *Kif1bp* is disrupted using CRISPR/Cas9. Our study confirms a role for KIF1BP in axon extension from subpopulations of peripheral and CNS neurons *in vivo*, but the smaller brain and olfactory bulbs we observed in *Kif1bp* null mutants suggest additional roles for KIF1BP during nervous system development.

## Methods

### Mouse strains

Two lines of *Kif1bp*^+/−^ mice were generated by CRISPR/Cas9 (see below). All experiments were approved by the Anatomy & Neuroscience, Pathology, Pharmacology and Physiology Animal Ethics Committee of the University of Melbourne and all experiments were performed in accordance with relevant guidelines and regulations. *Ret*^+/TGM^ mice, in which cDNA encoding tau-EGFP-myc (TGM) had been inserted into the first coding exon of Ret^12^, were mated to *Kif1bp*^+/−^ mice.

### Generation of Kif1bp null mutant mice using CRISPR/Cas

**Figure 1:**
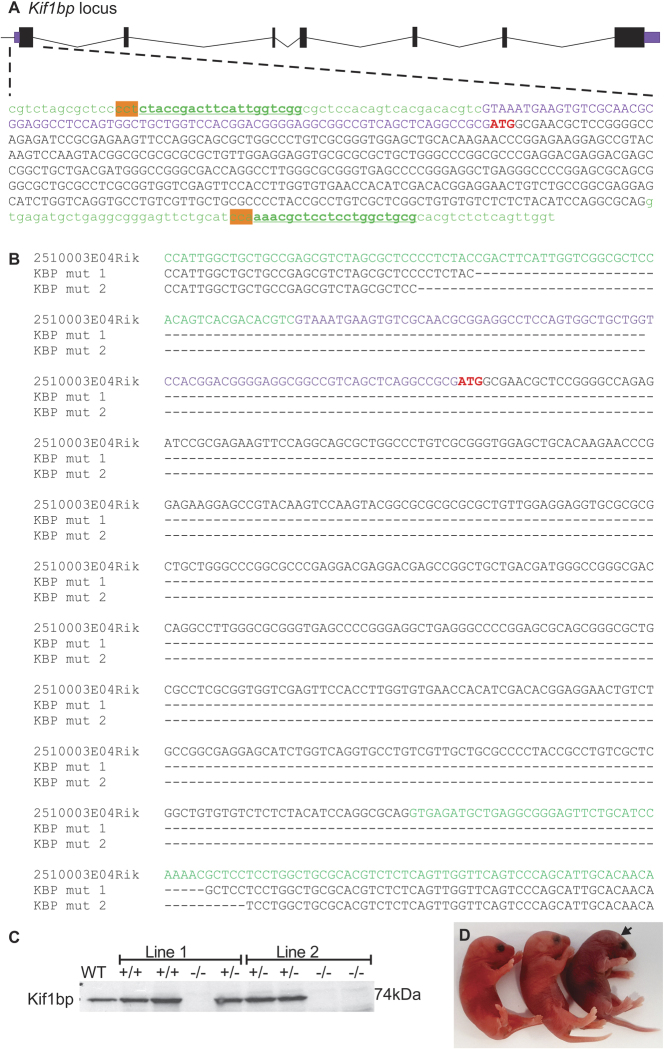
Generation of mice carrying mutations in *Kif1bp* generated by CRISPR-Cas9 editing. (**A**) Genomic structure of the *Kif1bp* locus showing exons (black boxes), UTR (purple), intronic DNA (green), the ATG start site (red), the sequence recognized by the RNA guides (bold and underlined) and the immediately adjacent PAM sequences (highlighted in orange). (**B**) Allelic sequences of wild-type (2510003E04Rik, as a reference) and the two heterozygote mutants (KBP mut 1 and KBP mut 2) from which the two lines were established. Hyphens show deleted sequences. (**C**) Western blot using an antibody directed against mKif1bp^6^ shows an absence of Kif1bp protein in null mutant mice in litters of pups from each of the two mouse lines. WT =  + / + , het =  +/− and CRISPR knockout = −/−. β-tubulin was used as a loading control. See Supplementary Figure 1 for full-length gels. (**D**) Three newborn pups from mating between *Kif1bp*^+/−^ mice. One pup (*arrow*) had cyanotic skin and was gasping, which genotyping revealed to be *Kif1bp*^−/−^.

Mice with targeted disruption of *Kif1bp* were generated by the Monash University Node of the Australian Phenomics Network. To delete exon 1 of mKBP (2510003E04Rik), guide RNA was designed to bind sequences flanking 5′UTR and intron 1–2. Guide RNA sequences, which were immediately followed by Protospacer Adjacent Motif (PAM) sequences, were cgaccaatgaagtcggtag (forward) and gcagccaggaggagcgttt (reverse) (Fig. 1A). After microinjection and transfer of two-cell stage embryos into a pseudopregnant female, the genotypes of the newborn pups were determined. The genomic regions flanking the gRNA target were amplified by PCR using specific primers: Fwd (5′CAGCGGAAGGCTCTGTATTC 3′) and Rev (5′TGATTCGGACGCTTAGGTTT 3′). Cloning and then sequencing identified two mutant mice where exon 1 was deleted. Although both mice were missing all of exon 1, the deletions were different (Fig. 1B). Hence, after confirmation of transmission of both mutations, two lines of *Kif1bp* mutant mice were established. Female and male heterozygous mutants for each line were mated.

### Genotyping of Kif1bp lines

Genotyping by PCR was performed on DNA extracted from tail tissues of newborn mice, or liver and tail from embryonic mice. Primers used for mKif1bp genotyping were as described above. PCRs were performed using standard PCR protocols with the addition of 5% DMSO in the reaction. Some genotyping of Kif1bp lines was also performed by Transnetyx (Cordova, TN, USA). Conditions and primers used to genotype *Ret*^TGM^ mice have been described previously^12^.

### Western blot

Protein was isolated from brain of E12.5 embryos generated by crossing mice heterozygous for *Kif1bp*. Tissue was lysed in RIPA buffer containing protease inhibitors, manually homogenised on ice and cleared by centrifugation at 14,000g at 4 °C for 15min before storing at −20C. Samples were processed further for SDS-PAGE followed by western blot analysis. Blots were blocked in 5% milk powder and incubated in either rabbit anti-Kif1bp (1:1000, kindly supplied by Maria Alves and Robert Hofstra) or mouse anti-E7 Tubulin (1:500, Developmental Studies Hybridoma Bank) at 4 °C overnight with constant agitation. The Kif1bp antibody was a rabbit polyclonal antibody directed against a peptide encompassing amino acids 24–38 of mKif1bp^6^. After washing in PBS/Tween 20, blots were incubated in either goat anti-rabbit IgG-HRP or goat anti-mouse IgG-HRP (Zymed, both 1:10,000). Bands were detected using chemiluminescence (Amersham ECL, GE Healthcare).

### Immunohistochemistry

Wholemount preparations of E12.5 gastrointestinal tract and lungs, E15.5 small intestine, E16.5 diaphragm and external muscle/myenteric plexus of P0 mice were fixed and processed for immunohistochemistry as described previously^13,14^. For cryosections of the olfactory bulb/nasal cavities and brainstems, newborn mice were injected with ketamine (200 mg/kg) and xylazine (20mg/kg) followed by transcardiac tissue perfusion with 4% paraformaldehyde. The heads were post-fixed overnight, washed and then cryoprotected. Cryosections of various tissues were processed for immunohistochemistry as described previously^15^ using the following primary antisera: goat anti-Sox10 (1:200, Santa Cruz, Dallas, Texas, USA), mouse anti-Tuj1 (1:2000, Covance, North Ryde, NSW, Australia), sheep anti-nNOS (1:2000, gift of Dr Piers Emson)^16,17^, human anti-HuC/D (1:5000, gift of Dr Vanda Lennon)^18^, sheep anti tyrosine hydroxylase (TH) (1:2000, Chemicon), guinea-pig anti-Phox2b (1:1000, gift of Prof. Hideki Enomoto)^19^, rabbit anti-NK1 receptor (NK1R) (1:1000, Millipore), goat anti-ChAT (1:50, Millipore) and rabbit anti-GABA (1:1000; Sigma). Secondary antisera used were donkey anti-sheep FITC (1:100, Jackson Labs), donkey anti-mouse Alexa594 (1:200, Molecular Probes), donkey anti-guinea pig FITC (1:100, Chemicon) and donkey anti-human Alexa594 (1:500, Jackson Labs). In some sections, nuclei were labelled with DAPI.

### Myenteric neuron counts in the distal colon of P0 mice

The number of Hu + and NOS + neurons present in stacked confocal images of 393 μm × 393 μm fields of view of wholemount preparations of distal colon were counted manually using Fuji cell counter.

### Measurement of migration distance

The distance from the ileocaecal junction to the most distal Sox10 + cell in wholemount preparations of E12.5 colon was measured from projected confocal images using Fuji software as described previously^20^. The distance from the junction between the stomach and small intestine and the most distal Sox10 + cell was also measured in wholemount preparations of gut from E10.5 mice using the same method.

### Measurement of area occupied by vagal fibers in the stomach and TH + fibers in the duodenum

Wholemount preparations of stomach from E12.5 mice were processed for Tuj1 immunostaining, imaged on a confocal microscope and then the area occupied by Tuj1 vagal branches measured in projected images using Fuji software. Wholemount E15.5 gut preparations were immunostained for tyrosine hydroxylase (TH), and then tiled confocal images were montaged into a single image. Using Fuji software, the first 2 mm of the duodenum was marked, and the area containing TH + fibers within the 2 mm zone was measured.

### Measurement of commissure thickness

P0 mice were perfusion fixed as described above and the brain processed for paraffin sectioning and Nissl staining. Sections were scanned and measurements taken using CaseViewer software. Both the anterior and posterior branches of the anterior commissure were measured. As many fibres in the anterior commissure of *Kif1bp*^*−/−*^ mice did not cross the midline, the thickness of the anterior commissure was measured between 200–300 μm from the midline. Measurements were collected across multiple sections and averaged. The thickness of the corpus callosum was measured at the midline at two points: Anteriorly at the level of the lateral septal nucleus, and posteriorly at the level of the ventral hippocampal commissure.

### Skull bone and cartilage staining

Skull bone and cartilage staining was performed using Alizarin Red and Alcian blue as described previously^21^.

### Statistics

GraphPad Prism 7.03 was used to analyse data and prepare graphs. All measurements were performed with the researcher blinded to the genotype of the preparations.

## Results

### Generation of Kif1bp null mutant mice using CRISPR/Cas technology

Using CRISPR-Cas9 editing, two lines of mice were generated in which exon 1 of *Kif1bp* was disrupted (Fig. 1A,B). Western blotting showed an absence of Kif1bp protein in newborn *Kif1bp*^−/−^ mice of both lines (Fig. 1C; see Supplementary Figure 1 for full-length gels). *Kif1bp*^−/−^ mice in both lines were born in the expected Mendelian ratios (20/75 for line 1 and 17/89 for line2), but died within 3–4 hours after birth. Most *Kif1bp*^−/−^ mice had cyanotic skin colour shortly after birth (Fig. 1C). Newborn *Kif1bp*^−/−^ mice also had significantly lower body weights than their littermates (1.20 ± 0.09 g for *Kif1bp*^−/−^ mice versus 1.31 ± 0.02 g for their littermates, mean ± S.D., unpaired t test, p = 0.0098). Breathing in newborn *Kif1bp*^−/−^ mice was characterized by sporadic deep gasping activities and an absence of detectable normal breathing movements (Supplementary movie). Histopathological analysis performed by the Australian Phenomics Network revealed that all major organs were present and there was no obvious pathology in the heart, thymus, lungs, trachea, diaphragm, pancreas, spleen, intestines or spinal cord. Alizarin Red and Alcian blue skull preparations did not reveal any noticeable defects in craniofacial morphology in P0 homozygous mutant mice (data not shown).

### Effects of loss of Kif1bp and interactions with Ret in the development of gut innervation

#### Intrinsic innervation

**Figure 2:**
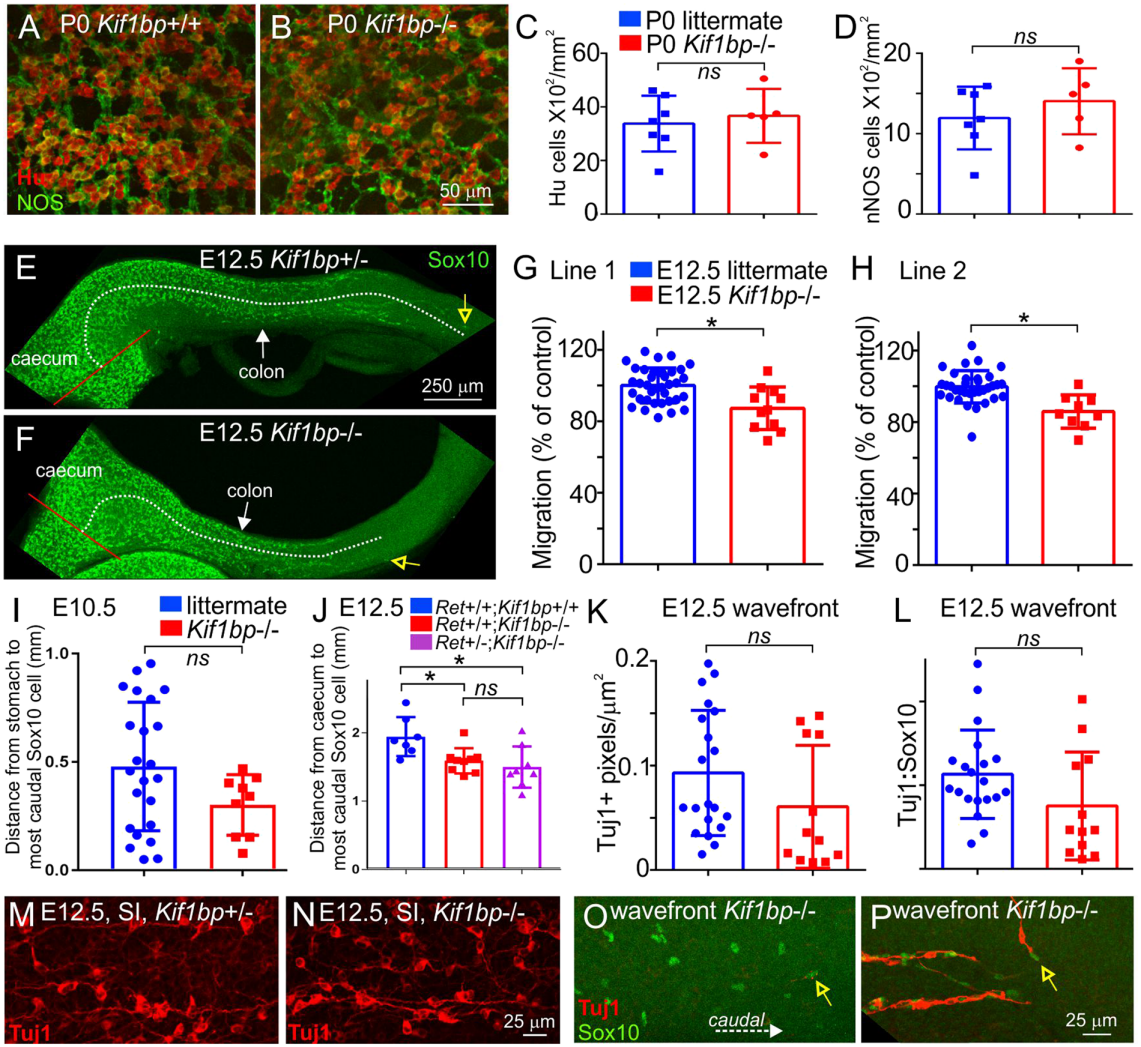
*Kif1bp* null mutants have delayed ENCC migration, but not distal aganglionosis or significant defects in enteric neurites. (**A**,**B**) Wholemount preparations of the external muscle layers of the rectum of newborn *Kif1bp*^+/+^ (**A**) and *Kif1bp*^−/−^ (B) mice after immunolabeling for the pan-neuronal marker, Hu (red), and the enteric neuron subtype marker, nNOS (green). (**C**,**D**) Densities of Hu + and nNOS + neurons (mean ± SD) in the myenteric plexus in line 1 of the *Kif1bp* mutants. There were no significant differences in the densities of Hu + (**C**) or nNOS + (**D**) myenteric neurons in the rectum (unpaired t tests, p > 0.05). (**E**,**F**) Wholemount preparations of colon from E12.5 *Kif1bp*^+/−^ (**E**) and *Kif1bp*^−/−^ (**F**) mice stained with antisera to Sox10. The most caudal Sox10 + cell in each preparation is shown by an open yellow arrow, the ileocaecal junction is indicated by a red line, and the distance from the ileocaecal junction to the most caudal Sox10 + cell by a white dotted line. (**G**,**H**) Quantification of the distance between the most caudal Sox10 + cell and the ileocaecal junction in E12.5 *Kif1bp*^−/−^ mice and their littermates (mean ± SD). Because of possible differences in age between litters, the distance from the ileocaecal junction to the most caudal Sox10 + cell in each preparation was expressed as a percentage of the mean distance from the ileocaecal junction to the most caudal Sox10 + in control (*Kif1bp*^+/+^ and *Kif1bp*^+/−^) preparations in that litter. The most caudal Sox10 + cell was significantly closer to the ileocaecal junction in *Kif1bp*^−/−^ mice compared to littermates in each line (unpaired t tests, p = 0.0008 for line 1 and p = 0.0002 for line 2). (**I**) There was no significant difference in the distance between the most caudal Sox10 + cell and the caudal end of the stomach in E10.5 *Kif1bp*^−/−^ mice and their littermates, and thus there does not appear to be a delay in the entry of vagal neural crest-derived cells into the gut. (**J**) *Kif1bp* does not appear to interact with *Ret* to regulate ENCC migration. The migration of ENCCs in E12.5 *Ret*^+/+^; *Kif1bp*^−/−^ mice was significantly delayed compared to *Ret*^+/+^; *Kif1bp*^+/+^ mice (ANOVA, p < 0.05), but there was no significant difference between E12.5 *Ret*^+/+^; *Kif1bp*^−/−^ and *Ret*^+/−^; *Kif1bp*^−/−^ mice (ANOVA). (**K**,**L**) The density of Tuj1 + neurites (number of Tuj1 + pixels/area) (**K**) p = 0.15) and the ratio of Tuj1 + pixels:Sox10 + cells (**L**) p = 0.08) at the migratory wavefront were not significantly different in E12.5 *Kif1bp*^−/−^ mice (red squares) from their littermates (blue circles). (**M**,**N**) Small intestine of E12.5 mice. The appearance of Tuj1 + neurites in E12.5 *Kif1bp*^−/−^ mice (**N**) was similar to that in littermates (**M**). (**O**,**P**) Migratory wavefront of ENCCs in the mid colon of E12.5 mice. The number of Tuj1 + neurites at the wavefront was very variable; in some E12.5 *Kif1bp*^−/−^ mice, there were very few neurites at and close to the wavefront (**O**), while in other E12.5 *Kif1bp*^−/−^ embryos, most Sox10 + ENCCs were associated with a neurite (**P**). The most caudal Sox10 + cell in each preparation is indicated with a yellow open arrow. All counts were performed blind to genotype.

Humans with recessive mutations in *KIF1BP* have GOSHS, and one of the characteristic malformations is Hirschsprung disease^4^. We therefore examined whether P0 *Kif1bp*^−/−^ mice have neurons along the entire length of the colon using antisera to the pan-neuronal marker, HuC/D, and the enteric neuron sub-type marker, nNOS. HuC/D + and nNOS + neurons were present along the entire colon in both lines of P0 *Kif1bp*^−/−^ mice (Fig. 2A,B). Furthermore, the densities of HuC/D and nNOS myenteric neurons were quantified in the distal colon of one line of P0 *Kif1bp*^−/−^ mice (line 1) and were not significantly different from littermates (Fig. 2C,D; unpaired t tests, p > 0.05). *Kif1bp* is expressed by the enteric nervous system in zebrafish^6^. RT-PCR (Supplementary Figure 2) and *in situ* hybridization (Supplementary Figure 3) studies confirmed expression of *Kif1bp* in ENCCs of embryonic mice, although gut mesenchymal and epithelial cells also expressed *Kif1bp* (Supplementary Figure 3).

Mutations in some genes result in delayed colonization of the gut by enteric neural crest-derived cells (ENCCs), but the entire gastrointestinal tract is eventually colonized^20,22^. We therefore examined whether the colonization of the gut by ENCCs is delayed in embryonic *Kif1bp*^−/−^ mice. Wholemount preparations of small and large intestine from E12.5 mice were processed for immunohistochemistry using the neural crest cell marker, Sox10, and the distance from the ileo-caecal junction to the most caudal Sox10 + cell measured; in E12.5 mice, the migratory wavefront in wild-type mice is in the mid-colon^23^. In both lines of mice, the migration of ENCCs was significantly delayed in E12.5 *Kif1bp*^−/−^ mice compared to littermate controls (Fig. 2E–H; unpaired t tests, p = 0.0008 for line 1 and p = 0.0002 for line 2). However, there was no significant difference in the distance from the stomach to the most caudal Sox10 + cell in E10.5 *Kif1bp*^−/−^ mice and their littermates (Fig. 2I), and thus there does not appear to be a delay in the entry of vagal neural crest-derived cells into the gut.

Mutations in *RET* are the most common cause of HSCR^24^, and *Ret*^+/−^ mice are genetically predisposed to HSCR-like distal aganglionosis^25–27^. We crossed *Kif1bp*^+/−^ mice (line 1) with *Ret*^+/TGM^ mice, and then inter-crossed *Kif1bp*^+/−^; *Ret*^+/TGM^ mice. In confirmation with the data shown in Fig. 2G,H, the migration of ENCCs in E12.5 *Ret*^+/+^; *Kif1bp*^−/−^ mice was significantly delayed compared to *Ret*^+/+^; *Kif1bp*^+/+^ mice (ANOVA, p < 0.05), but there was no significant difference between E12.5 *Ret*^+/+^; *Kif1bp*^−/−^ and *Ret*^+/TGM^; *Kif1bp*^−/−^ mice (Fig. 2J; ANOVA). Thus *Kif1bp* does not appear to interact with *Ret* to regulate ENCC migration.

*kif1bp* mutant zebrafish exhibit delayed and defective axon growth in the peripheral nervous system, including the ENS^7^. ENCCs migrate in close association with neurites of enteric neurons^28,29^. At mid-embryonic ages, most neurites project caudally along the longitudinal axis of the gut^29,30^. We used Tuj1 antisera to examine neurites of intrinsic neurons in the gut of E12.5 mice. In the small intestine of both *Kif1bp*^+/+^ and *Kif1bp*^+/−^ mice, many Tuj1 + neurites projected caudally, and the appearance of the neuronal network was not obviously different between genotypes (Fig. 2M). We also examined Tuj1 + neurites associated with ENCCs at the migratory wavefront in the mid-colon of E12.5 mice and quantified the density of Tuj1 staining in the same field of view as the most caudal Sox10 + cell. The number of Tuj1 neurites at the migratory wavefront was very variable between embryos of all genotypes; in some embryos there were very few Tuj1 + neurites associated with the most caudal Sox10 + ENCCs (Fig. 2O), while in other embryos, most ENCCs were associated with Tuj1 + neurites (Fig. 2P). Although the four lowest values for the density of Tuj1 + pixels (number of Tuj1 + pixels/area of gut) at the migratory wavefront were all from *Kif1bp*^−/−^ mice, overall there was no significant difference in the density of Tuj1 + pixels between *Kif1bp*^−/−^ mice and their littermates (Fig. 2K, unpaired t test, p = 0.15). There were also no significant differences in the density of Sox10 + cells close to the migratory wavefront (data not shown, unpaired t test, p = 0.4) or in the ratio of Tuj1 + neurites:Sox10 + ENCCs (Fig. 2L, unpaired t test, p = 0.08), although the four lowest values for the Tuj1:Sox10 ratio were from *Kif1bp*^−/−^ mice. Hence we are unable to associate the delay in ENCC migration with defects in neurites close to the migratory wavefront.

#### Extrinsic innervation of the gut

**Figure 3:**
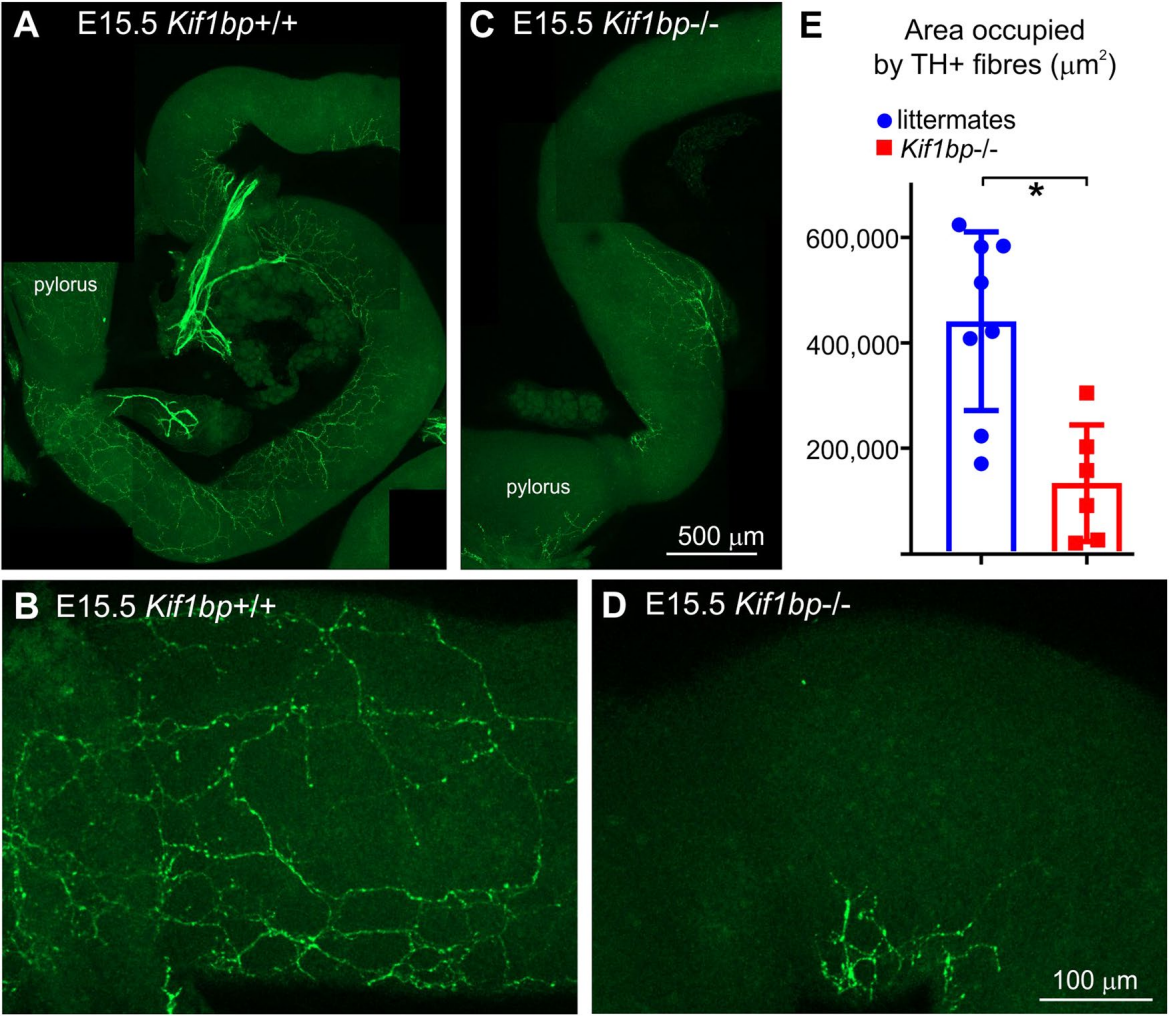
*Kif1bp* null mutants have delayed growth of sympathetic fibers into the small intestine. Low (**A**,**C**) and higher (**B**,**D**) magnification images of wholemount preparations of the duodenum of E15.5 *Kif1bp*^+/+^ (**A**,**B**) and *Kif1bp*^−/−^ (**C**,**D**) mice after immunolabeling for tyrosine hydroxylase (TH, green). (**E**) The area occupied by TH + fibers in the first 2 mm of small intestine (mean ± SD) was significantly less in E15.5 *Kif1bp*^−/−^ mice compared to their littermates (unpaired t test, p = 0.002).

The axons of sympathetic neurons that innervate the intestines project along intestinal arteries and first enter the small intestine around E15.5^31^. The area occupied by tyrosine hydroxylase (TH) + fibers in the first 2 mm of duodenum in E15.5 *Kif1bp*^+/+^ and *Kif1bp*^−/−^ mice was compared (Fig. 3A–D), and was significantly smaller in *Kif1bp*^−/−^ mice (Fig. 3E, unpaired t test, p = 0.002).

**Figure 4:**
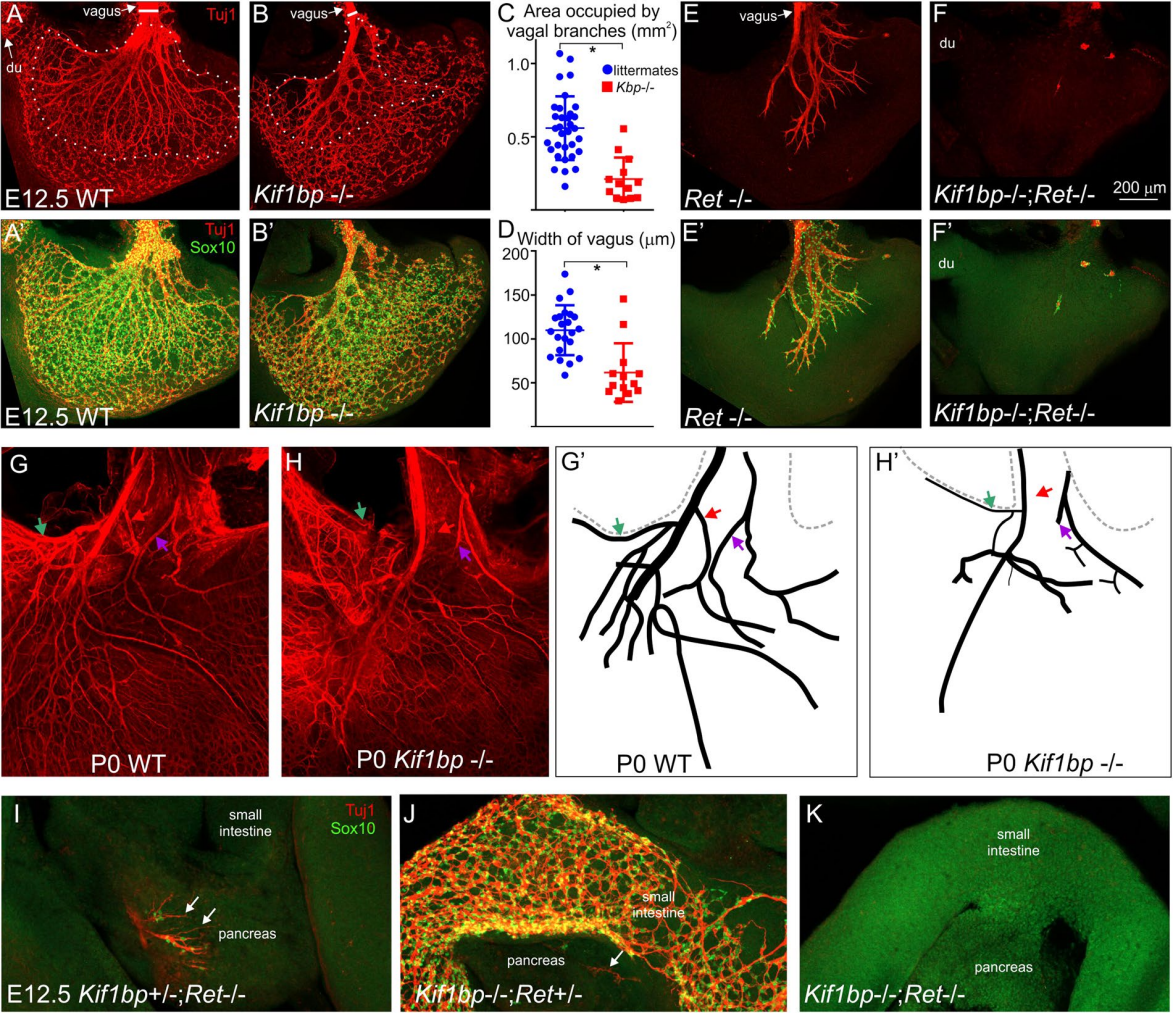
*Kif1bp* is required for the development of the vagal innervation of the stomach and pancreas, and interacts with *Ret*. (**A**,**A’**,**B**,**B’**). Wholemount preparations of the stomach of E12.5 *Kif1bp*^+/+^ (**A**,**A’**) and *Kif1bp*^−/−^ (**B**,**B’**) mice after immunolabeling for the pan-neuronal marker, Tuj1(red), and Sox10 (green), which labels all non-neuronal ENCCs. (**C**,**D**) The area occupied by Tuj1 + vagal branches (see dotted lines in **A**,**B**) and the width of the vagus nerve at the gastroesophageal junction (see horizontal white line in **A**,**B**) were quantified. The area occupied by vagal nerve fibers (**C**) and the width of the vagus nerve (**D**) were significantly smaller in E12.5 *Kif1bp*^−/−^ mice compared to their littermates. (**E-F**). *Kif1bp* interacts with *Ret* to regulate the development of the vagal innervation of the stomach. In the stomach of E12.5 *Kif1bp*^+/+^; *Ret*^TGM/TGM^ mice, Sox10 + cells and Tuj1 + vagal nerve fibers were present only in the proximal stomach, and all Sox10 + cells were associated with vagal nerve fibers (**E**,**E’**). In most *Kif1bp*^−/−^; *Ret*^TGM/TGM^ mice, vagal nerve fibers were absent from the stomach, and the only Tuj1 staining was associated with a very small number of intrinsic neurons (**F**,**F’**). (**G**,**H**) The vagal innervation of the stomach is still defective at birth. Tuj1 staining (**G**,**H**) and drawings (**G’H’**) of the same wholemount preparations of P0 wildtype (**G**,**G’**) and *Kif1bp*^−/−^ (**H**,**H’**) mice. In newborn *Kif1bp*^−/−^ mice, some vagal branches are thinner (*green arrow*), missing (*red arrow*) or shorter (*purple arrow*). (**I**–**K**) Wholemount preparations of E12.5 small intestine and pancreas after immunolabeling for Tuj1(red) and Sox10 (green). Tuj1 + vagal fibers (*arrows*) are present in the pancreas of *Kif1bp*^+/−^; *Ret*^TGM/TGM^ mice (**I**) and in small numbers in *Kif1bp*^−/−^; *Ret*^+/TGM^ mice (**J**), but are absent in *Kif1bp*^−/−^; *Ret*^TGM/TGM^ mice (**K**). Note the absence of Sox10 + ENCC within the small intestine of *Kif1bp*^+/−^; *Ret*^TGM/TGM^ and *Kif1bp*^−/−^; *Ret*^TGM/TGM^ mice (**I**,**K**), but Sox10 + ENCC are present within the small intestine of *Kif1bp*^−/−^; *Ret*^+/TGM^ mice (**J**). The Sox10 + cells (green) associated with the vagal fibres in the pancreas are likely to be Schwann cell precursors.

The vagal innervation of the stomach was examined using Tuj1 antisera, which labels intrinsic neurons as well as vagal fibers. At the gastroesophageal junction of E12.5 *Kif1bp*^+/+^ mice, large bundles of vagal nerve fibers extended into the stomach and then branched into smaller bundles. The area occupied by branches of the vagus was significantly smaller in E12.5 *Kif1bp*^−/−^ mice compared to their littermates (Fig. 4A–C, unpaired t test, p < 0.0001), and vagal branches were often missing in the proximal stomach of the null mutants (Fig. 4B). The width of the vagus nerve at the gastroesophageal junction was also smaller in E12.5 *Kif1bp*^−/−^ mice (Fig. 4A,B,D, unpaired t test, p < 0.0001). However, the defect was not fully penetrant as the vagal innervation of the stomach of two *Kif1bp*^−/−^ mice was well within the normal range (Fig. 4C,D). Vagal branches were also missing, smaller or shorter in the stomach of newborn *Kif1bp*^−/−^ mice (Fig. 4G,H).

*Ret* null mutant mice do not have any neurons in the small and large intestine, and in the stomach, neurons are only present in the proximal stomach^32,33^. In the stomach of E12.5 *Kif1bp*^+/+^-; *Ret*^+/+^ and *Kif1bp*^+/−^; *Ret*^+/TGM^ mice, Sox10 + cells and Tuj1 + vagal nerve fibers were present throughout the stomach. In E12.5 *Kif1bp*^+/+^; *Ret*^TGM/TGM^ and *Kif1bp*^+/−^; *Ret*^TGM/TGM^ mice, Sox10 + cells and Tuj1 + vagal nerve fibers were present only in the proximal stomach, and all Sox10 + cells were associated with vagal nerve fibers (Fig. 4E,E’). In 2/4 *Kif1bp*^−/−^; *Ret*^TGM/TGM^ mice, vagal nerve fibers were absent from both sides of the stomach, and the only Tuj1 staining was associated with a very small number of intrinsic neurons (Fig. 4F,F’); in the other 2/4 *Kif1bp*^−/−^; *Ret*^TGM/TGM^ embryos examined, a small number of short vagal fibers was present on one side of the stomach only. No vagal fibers were observed in the vicinity of the pancreas in double mutants (Fig. 4K), but were present in other phenotypes (Fig. 4I,J). Thus *Kif1bp* interacts with *Ret* to regulate the development of the vagal innervation of the gut. In *Kif1bp*^−/−^; *Ret*^TGM/TGM^ mice, all Sox10 + cells in the esophagus appeared to be associated with vagal nerve fibers.

### Effects of loss of Kif1bp on the development of the innervation of the lungs and diaphragm

**Figure 5:**
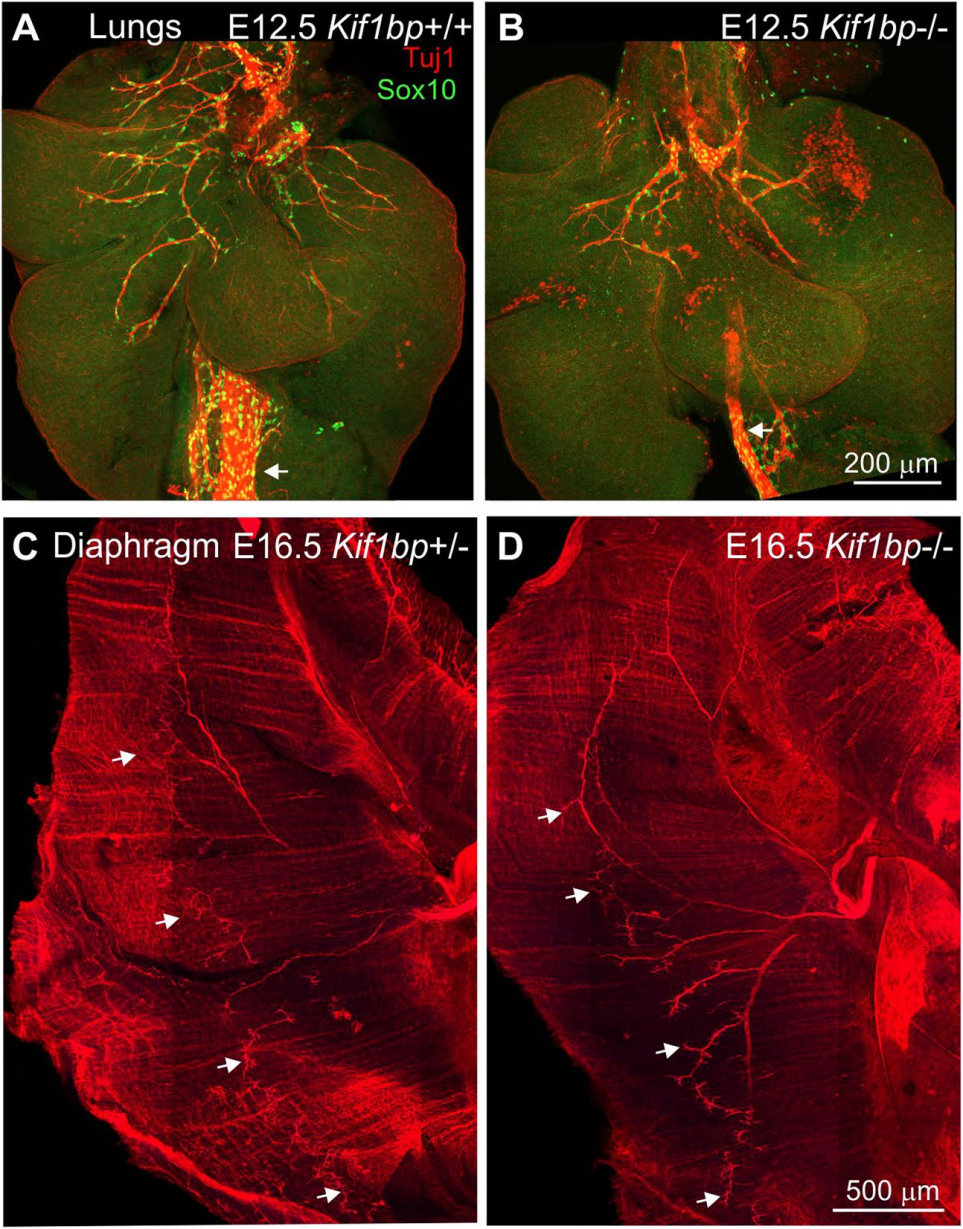
Loss of *Kif1bp* results in decreased vagal fibers in the lungs but does not affect the development of the phrenic innervation to the diaphragm. (**A**,**B**). Wholemount preparations of lungs of E12.5 *Kif1bp*^+/+^ (**A**) and *Kif1bp*^−/−^ (**B**) mice following processing for Tuj1 immunostaining. There are reduced numbers of vagal fibers in the lungs of *Kif1bp*^−/−^ mice. Note also the smaller width of the vagus nerve running next to the esophagus (*arrows*) in the null mutant. (**C**,**D**). Wholemount preparations of costal muscle of the diaphragm of E16.5 *Kif1bp*^+/−^ (**C**) and *Kif1bp*^−/−^ (**D**) mice following processing for Tuj1 immunostaining. In *Kif1bp*^−/−^ mice, Tuj1+ nerve terminals (*arrows*) are present in similar numbers and appearance to *Kif1bp*^+/+^ and *Kif1bp*^+/−^ mice.

Because *Kif1bp*^−/−^ mice die very shortly after birth and exhibit breathing defects the innervation of the lungs and diaphragm were examined. The number of Tuj1 + (vagal) fibers in the lungs of E12.5 *Kif1bp*^−/−^ mice was reduced (Fig. 5A,B). However, there were no noticeable differences in the phrenic innervation of the diaphragm of E16.5 *Kif1bp*^−/−^ mice compared to littermates (Fig. 5C,D).

### Effects of loss of Kif1bp on the brain

#### Brain size and olfactory bulbs

**Figure 6:**
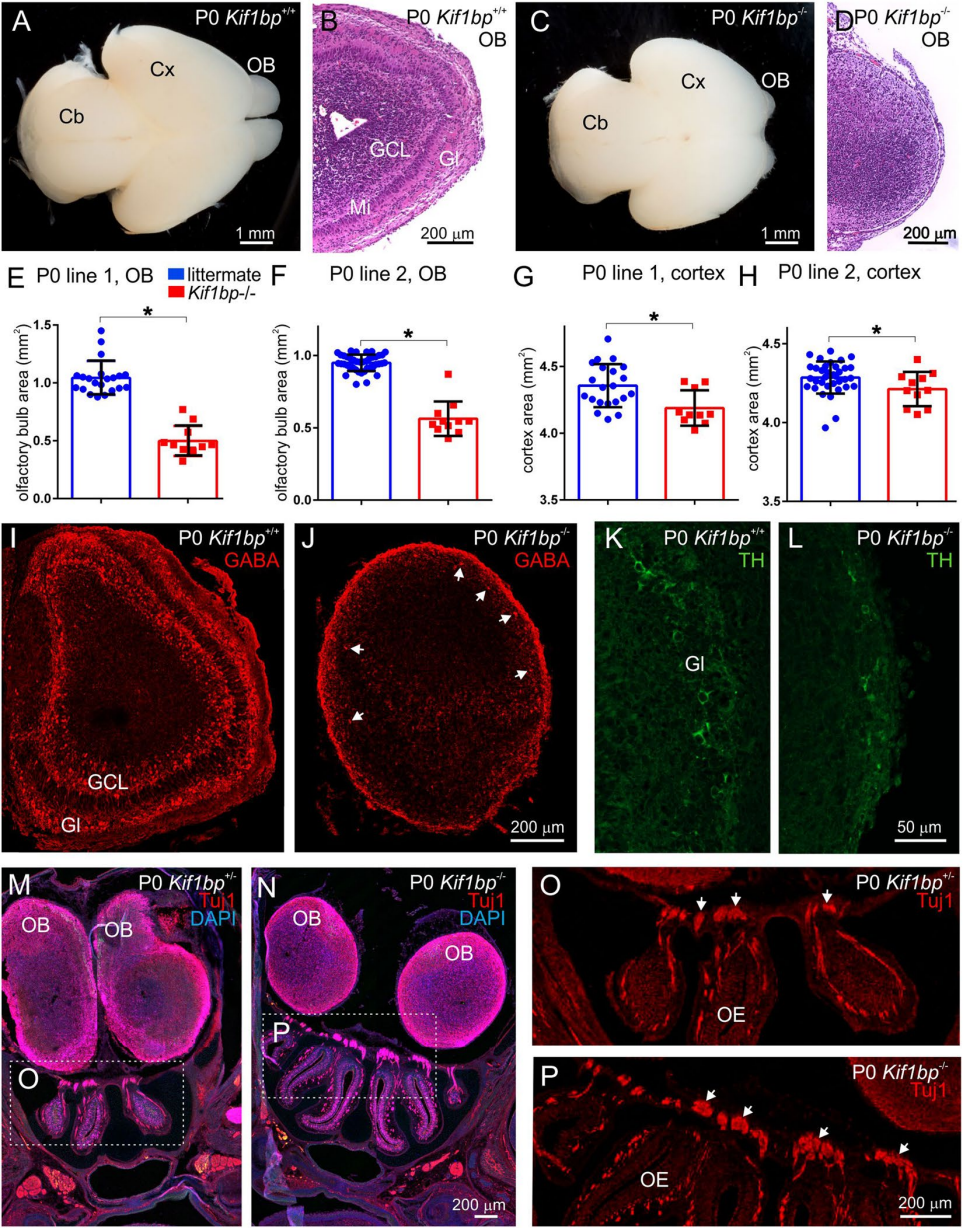
*Kif1bp* null mutants have smaller olfactory bulbs and cerebral cortices. (**A**,**C**) Dorsal view of brains of newborn wildtype (**A**) and *Kif1bp*^−/−^ (**C**) mice. Note the smaller olfactory bulbs (*OB*) in the *Kif1bp*^−/−^ mouse (**C**). *Cx* – cerebral cortex; *Cb* – cerebellum. (**B**,**D**) Horizontal sections through the olfactory bulbs of newborn wildtype (**B**) and *Kif1bp*^−/−^ (**D**) mice stained for haemotoxylin and eosin. The layers of the olfactory bulb, including the glomerular layer (*Gl*), mitral cell layer (*Mi*) and granule cell layer (*GCL*) are apparent in the wildtype (**B**), but not the null mutant (**D**). Quantification of the size of the olfactory bulbs (**E,F**) and cerebral cortex (**G,H**) in both lines of *Kif1bp* mutant mice determined from photographs of the dorsal views of brains. (**E**,**F**) In both lines of mice, the olfactory bulb is significantly smaller in area in *Kif1bp*^−/−^ mice (unpaired t tests, p = 0.0001 for both lines). (**G**,**H**) In both lines, there is also a smaller, but significant, difference in the area of the cortex (p = 0.008 for line 1 and p = 0.048 for line 2). Note that the y axis does not start at 0 for the graphs of cortical area. (**I**–**L**) Coronal sections through the olfactory bulb stained using antibodies to GABA (**I,J**) and tyrosine hydroxylase (TH, **K,L**). (**I**) GABA neurons are abundant in the glomerular layer (*Gl*) and granule cell layer (*GCL*) in the olfactory bulb of the wildtype mouse. (**J**) In the *Kif1bp* null mutant, there are only sparse GABA + neurons in the outer regions of the olfactory bulb (*arrows*). (**K**) TH-immunoreactive neurons are present in the glomerular layer (Gl) of the wildtype mouse. (**L**) TH + neurons were also present in the outer regions of the olfactory bulb of *Kif1bp*^−/−^ mice, but were sparse and did not form a continuous layer. (**M**,**N**) Coronal sections through the nasal cavities and olfactory bulbs of *Kif1bp*^+/−^ (**M**) and *Kif1bp*^−/−^ (**N**) mice immunostained using an antibody to Tuj1 and counterstained with DAPI. *OB* – olfactory bulb. (**O**,**P**) Higher magnification images of the olfactory epithelium (indicated by the dotted boxes in **M** and **N**) showing Tju1 + axons projecting from the olfactory epithelium towards the olfactory bulbs in both *Kif1bp*^+/−^ and *Kif1bp*^−/−^ mice.

Newborn *Kif1bp*^−/−^ mice had significantly lower brain weights than their siblings (0.09 ± 0.01 g for *Kif1bp*^−/−^ mice versus 0.11 ± 0.01 g for their littermates, mean ± S.D., unpaired t test, p = 0.01). Inspection of the brains of newborn *Kif1bp*^−/−^ mice revealed noticeably smaller olfactory bulbs (Fig. 6A,C). This was confirmed by measuring the area occupied by the olfactory bulbs from photographs of the dorsal aspect of the brain of P0 mice; in both lines of *Kif1bp* mice, the olfactory bulbs of *Kif1bp*^−/−^ mice were about 50% smaller than *Kif1bp*^+/+^ and *Kif1bp*^+/−^ littermates (Fig. 6E,F, unpaired t tests, p = 0.0001 for both lines). The cortical surface area was also slightly, but significantly, smaller in *Kif1bp*^−/−^ mice (Fig. 6G,H, p = 0.008 for line 1 and p = 0.048 for line 2). Histological analysis revealed that the olfactory bulbs of newborn *Kif1bp*^−/−^ mice were not laminated (Fig. 6B,D), while immunohistochemical studies showed that GABA + and TH + neurons were present in the olfactory bulb of *Kif1bp* null mutants, but were sparse and were only found close to the periphery (Fig. 6I–L). There was no obvious difference in the appearance of Tuj1 + axons projecting from the olfactory epithelium towards the olfactory lobes in horizontal sections of P0 mice (Fig. 6M–P).

#### White matter tracts

**Figure 7:**
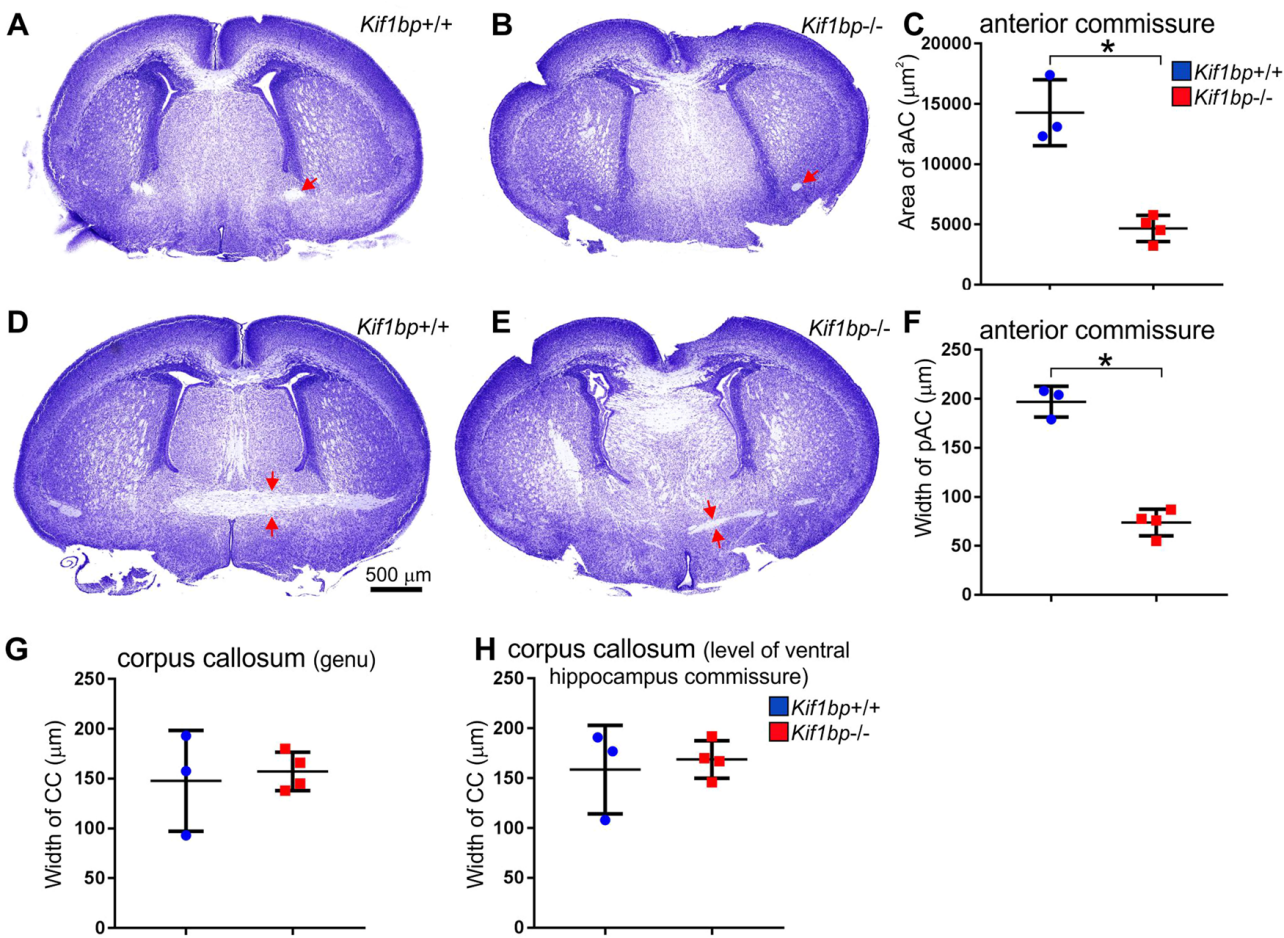
*Kif1bp* null mutants have defects in the anterior commissure (AC). (**A**,**B**,**D**,**E**) Nissl stained, coronal sections of P0 mice, at the levels of the genu of the corpus callosum (**A,B**) and the ventral hippocampal commissure (**D,E**) showing the AC (*red arrows*). The anterior AC (aAC) and posterior AC (pAC) are significantly smaller in *Kif1bp*^−/−^ mice than wild-type mice (**C**,**F**) (unpaired t tests, p = 0.0013 and p = 0.0001). (**G**,**H**) There was no significant difference in the width of the corpus callosum at the same levels (p = 0.7 and p = 0.69).

The width of the corpus callosum and anterior commissure (ac), two of the major white matter tracts in the brain were quantified in Nissl-stained coronal sections. The width of the pars anterior ac and the more posterior ac were measured 200–300 μm from the midline, and were significantly thinner in *Kif1bp*^−/−^ mice (Fig. 7A–F, unpaired t tests, p = 0.0013 and p = 0.0001). In the mutants, very few fibers crossed the midline, but appeared to divert dorsally, possibly with fibers in the fornix. However, there was no significant difference in the width of the corpus callosum measured at the midline at level of the lateral septal nucleus or more posteriorly at the level of the ventral hippocampal commissure (Fig. 7G,H, p = 0.7 and p = 0.69).

#### Respiratory nuclei in the brainstem

**Figure 8:**
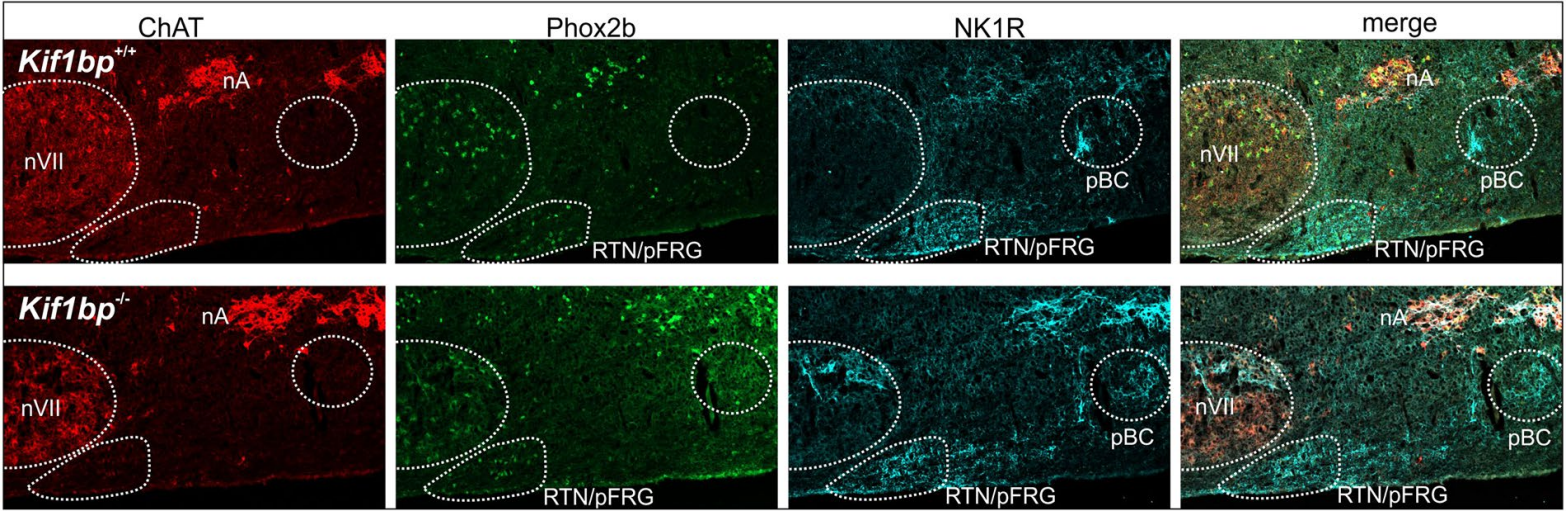
No obvious defects in the respiratory nuclei in the brainstem of *Kif1bp* null mutants. Sagittal cryosections of the brainstem of P0 mice immunostained using antisera to ChAT (red), Phox2b (green) and the neurokinin 1 receptor (NK1R, blue). Phox2b + staining of the retrotrapezoid nucleus/parafacial respiratory group (RTN/pFRG) and NK1R staining of the pre-Bötzinger complex (pBC) did not reveal any noticeable differences between P0 wild-type mice (top panels) and *Kif1bp*^−/−^ mice (bottom panels).

Mice harbouring human mutations in *PHOX2B* associated with congenital hypoventilation syndrome (CCHS) die perinatally from breathing defects, and some of the respiratory nuclei in the brainstem are missing^34^. Because *Kif1bp*^−/−^ mice exhibit breathing, defects, we examined the major nuclei in sagittal sections of the brainstem of newborn mice using antisera to Phox2b, choline acetylcholine transferase (ChAT) and the neurokinin-1 receptor. All major brainstem motor nuclei were present in P0 *Kif1bp*^−/−^ mice including the facial (Fig. 8), trigeminal and hypoglossal nuclei as well as the ambiguus nucleus (Fig. 8) and dorsal motor nucleus of the vagus nerve. Moreover, the primary respiratory phase oscillators such as the retrotrapezoid nucleus/parafacial respiratory group, revealed by Phox2b staining^35,36^ and the pre-Bötzinger complex, revealed by neurokinin 1 receptor staining^37,38^, were also present and not noticeably different in size from wild-type mice (Fig. 8).

## Discussion

We generated *Kif1bp* null mice using Crispr/Cas9. Both mouse lines generated showed the same phenotype of neonatal death, decreased brain size, including cerebral cortex, smaller and disorganised olfactory bulbs, reduced anterior commissure, delayed enteric neural crest colonisation of the gut and defective vagal and sympathetic innervation of the gut. We did not observe craniofacial abnormalities or HSCR in the mouse model. These latter aspects of the phenotype are similar to *kif1bp* mutant zebrafish, which displayed reduced axonal growth, but neurons were present along the length of the gut and no craniofacial abnormalities were observed^7^.

Similarities between the mouse model and GOSHS in humans include alterations in brain development, particularly a reduced overall size of the brain. The reduced size of the olfactory bulbs in mutant mice was particularly striking. The olfactory bulbs also lacked normal lamellar organisation, although at least some of the normal neuronal subtypes were present. Moreover, a significant decrease in size of the anterior commissure was observed. Because the two olfactory bulbs are interconnected via the anterior limb of the anterior commissure, the reduction in anterior commissure size may be due to the reduction in olfactory bulb size. Further, the reduced development of the olfactory bulb may decrease output from the olfactory bulb to the olfactory cortex and in turn result in reduced communication between olfactory cortices, which normally communicate via axons in the posterior limb of the anterior commissure^39^. In one reported case of GOSHS, MRI showed an absence of the anterior commissure^40^, however this patient was not genetically confirmed to have GOSHS, and may have had the phenotypically similar but genetically distinct Mowat-Wilson syndrome instead. We were unable to find any reports of defective sense of smell in GOSHS patients, although smell is not routinely included in neurological assessments, and smell identification tests would be difficult to perform in GOSHS patients, who also have mental retardation. Like GOSHS in humans, most of the defects we observed in *Kif1bp*^−/−^ mice were not fully penetrant.

Most GOSHS patients have Hirschsprung disease^4^. *Kif1bp* mutant mice displayed delayed enteric neural crest colonisation of the gut at E12.5, however by birth the entire gastrointestinal tract was colonised and the mice therefore did not display a Hirschsprung disease phenotype. Zebrafish with mutant *kif1bp* also have full colonisation of the gut^7^. The human gastrointestinal tract is much longer than that of mouse and zebrafish, and hence human enteric neural crest cells must migrate substantially further than in smaller animals. The human gut may therefore be more sensitive to delays in neural crest migration. Differences between human patients and other mouse models of HSCR support this idea. For example, heterozygous mutations of *RET* can be sufficient to cause HSCR in humans^24^, while *Ret*^*+/−*^ mice do not have HSCR-like aganglionosis^41^. However, reducing the amount of Ret in mice to approximately one third of wild-type levels does cause HSCR-like aganglionosis^42^.

In mice, most enteric neural crest-derived cells migrate in close association with neurites^28,29^. We examined if the delay in migration of enteric neural crest cells in *Kif1bp* mutants might be due to a reduction in the number of neurites along the migratory pathway. However, there was no significant difference in the amount of Tuj1 staining close to the migratory wavefront in *Kif1bp* null mutants, and so the mechanism by which Kif1bp regulates enteric neural crest cell migration remains to be determined.

Mice lacking Kif1bp exhibited some phenotypic features that have not been reported in GOSHS patients, including defects in the extrinsic innervation of the bowel. Previous studies have shown that the development of the vagal innervation of the mouse stomach requires netrin-1^43^, Slit ligands^44^, BDNF^45^, and the transcription factor, Tbx1^46^, while artemin^47^, NGF^48^ and netrin-1^49^ play a role in the development of the sympathetic innervation of the gut^50^. Our data show that Kif1bp is required for the development of the vagal innervation of the stomach and the sympathetic innervation of the intestine. *kif1bp* mutant zebrafish were reported to have reduced axons in the ENS, although it was not determined whether intrinsic and/or extrinsic neurons were affected^7^. Sensory axonal neuropathy has been reported in one case of GOSHS^3^, but to our knowledge gastric motility and secretion have not been examined in patients with GOSHS. In future studies it would be interesting to examine vagal and sympathetic ganglia in *Kif1bp* mutants to determine if the reduced innervation is due to reduced numbers of cell bodies and/or defects in axon projections.

We observed an interaction between *Kif1bp* and *Ret* in the development of vagal innervation of the stomach, but not in the colonization of the intestine by neural crest-derived cells. In mice lacking only Ret, vagal nerve fibers occupied around one third of the stomach surface, whereas in mice lacking both Ret and Kif1bp, there were almost no vagal nerve fibers in the stomach or projecting to the pancreas.

An incidental observation of our study was that in *Ret*^−/−^ mice, almost all of the Sox10 + cells, which are only present in the proximal stomach, were associated with vagal nerve fibers. Recent studies have shown that peripheral glial stem cells (also called Schwann cell precursors) are the source of parasympathetic neurons^51,52^, sub-populations of enteric neurons^53,54^ and adrenal chromaffin cells^55^. Our data support a very recent study showing that enteric neurons in the esophagus and stomach arise from Schwann cell precursors that navigate into the esophagus and stomach along the vagus nerve^53^.

In the current study, loss of Kif1bp only affected a sub-population of axons in the peripheral and central nervous systems of mice. For example, we found no evidence that the axons of enteric neurons, the phrenic nerve innervating the diaphragm or the corpus callosum were affected in *Kif1bp* null mutants. The mechanism by which Kif1bp affects axonal outgrowth and transport has been examined in several studies. A yeast two-hybrid screen using an E11 mouse cDNA library showed that KIF1BP binds to microtubule-associated proteins^6^, and in human fibroblasts, KIF1BP was reported to co-localize and interact with α-tubulin and F-actin^2^. Moreover, recent studies have demonstrated that KIF1BP binds to and regulates a variety of kinesin motor proteins involved in microtubule cytoskeletal organization and neuronal cargo transport^9,11^. KIF1BP has been shown to bind KIF1B, SCG10^6,11^, KIF1A, KIF1C, KIF3A, KIF13B, KIF14, KIF15 and KIF18A^9^, however no association was detected between SCG10 and KIF1BP in the latter study. These members of the kinesin superfamily are from different subfamilies, which have different functions and transport different cargo^56^. As KIF1BP can bind to multiple different members of these subfamilies, it is possible that the differential effects of loss of Kif1bp on axon projections from different neuronal populations might reflect differences in the expression of particular KIFs by different neuron types.

Unlike GOSHS patients, *Kif1bp* null mutant mice died within several hours of birth. Premature death was associated with respiratory failure, as indicated by cyanosis and hypoxic gasping. It is possible that respiratory failure is linked to reduced vagal innervation of the lungs. Vagal feedback linked to the Hering-Breuer reflex of pulmonary stretch receptors^57^ is critical for neonatal breathing^58^, and vagotomy triggers acute respiratory failure in neonates^59^. Thus reduced vagal innervation of the lung may be a major contributing factor for respiratory failure observed in *Kif1bp*^−/−^ mice. Although critical respiratory centers and motor nuclei in the brainstem appear to be normally developed and the diaphragm is innervated in *Kif1bp* null mutants, we also cannot rule out the possibility of defects in the connectivity of the neural circuitry underlying respiration. It would be interesting to restore Kif1bp only in the nervous system to determine if neonatal lethality is rescued.

To date, the main cellular functions identified for KIF1BP are to regulate axon growth and synaptic vesicle transport^6,7,9,11^. However, our data showing reduced olfactory bulb and brain size strongly suggest that KIF1BP must also play an essential role in survival, proliferation and/or neuronal differentiation during development of some parts of the nervous system.

In conclusion, we have generated the first mouse model of GOSHS, which exhibit defects in the development of subpopulations of CNS and peripheral neurons. It is not surprising that mutations that affect general developmental neurobiological processes such as axon extension (this study) and synapse development that underlie autism^60^ affect both central and peripheral neurons.

## Electronic supplementary material

Supplementary information

video of newborn Kif1bp mice

## References

[1] Brooks AS (1999). A consanguineous family with Hirschsprung disease, microcephaly, and mental retardation (Goldberg-Shprintzen syndrome). J Med Genet.

[2] Drevillon L (2013). KBP-cytoskeleton interactions underlie developmental anomalies in Goldberg-Shprintzen syndrome. Hum Mol Genet.

[3] Dafsari HS (2015). Goldberg-Shprintzen megacolon syndrome with associated sensory motor axonal neuropathy. Am J Med Genet A.

[4] Brooks, A.S. & Hofstra, R.M.W. KIAA1279 and Goldberg-Shprintzen Syndrome, In *Epstein’s Inborn Errors of Development: The Molecular Basis of Clinical Disorders of Morphogenesis*. (eds. Erickson, R. P. & Wynshaw-Boris, A. J.) 1417–1421 (Oxford University Press, Oxford; 2016).

[5] Brooks AS (2005). Homozygous nonsense mutations in KIAA1279 are associated with malformations of the central and enteric nervous systems. Am J Hum Genet.

[6] Alves MM (2010). KBP interacts with SCG10, linking Goldberg-Shprintzen syndrome to microtubule dynamics and neuronal differentiation. Hum Mol Genet.

[7] Lyons DA, Naylor SG, Mercurio S, Dominguez C, Talbot WS (2008). KBP is essential for axonal structure, outgrowth and maintenance in zebrafish, providing insight into the cellular basis of Goldberg-Shprintzen syndrome. Development (Cambridge, England).

[8] Salehpour S, Hashemi-Gorji F, Soltani Z, Ghafouri-Fard S, Miryounesi M (2017). Association of a Novel Nonsense Mutation in KIAA1279 with Goldberg-Shprintzen Syndrome. Iranian journal of child neurology.

[9] Kevenaar JT (2016). Kinesin-Binding Protein Controls Microtubule Dynamics and Cargo Trafficking by Regulating Kinesin Motor Activity. Curr Biol.

[10] Wozniak MJ, Melzer M, Dorner C, Haring HU, Lammers R (2005). The novel protein KBP regulates mitochondria localization by interaction with a kinesin-like protein. BMC Cell Biol.

[11] Drerup CM, Lusk S, Nechiporuk A (2016). Kif1B Interacts with KBP to Promote Axon Elongation by Localizing a Microtubule Regulator to Growth Cones. J Neurosci.

[12] Enomoto H (2001). RET signaling is essential for migration, axonal growth and axon guidance of developing sympathetic neurons. Development (Cambridge, England).

[13] Hao MM (2010). The role of neural activity in the migration and differentiation of enteric neuron precursors. Neurogastroenterol Motil.

[14] Hotta R (2013). Transplanted progenitors generate functional enteric neurons in the postnatal colon. The Journal of clinical investigation.

[15] McKeown SJ, Mohsenipour M, Bergner AJ, Young HM, Stamp LA (2017). Exposure to GDNF Enhances the Ability of Enteric Neural Progenitors to Generate an Enteric Nervous System. Stem cell reports.

[16] Herbison AE, Simonian SX, Norris PJ, Emson PC (1996). Relationship of neuronal nitric oxide synthase immunoreactivity to GnRH neurons in the ovariectomized and intact female rat. J Neuroendocrinol.

[17] Young HM, Ciampoli D (1998). Transient expression of neuronal nitric oxide synthase by neurons of the submucous plexus of the mouse small intestine. Cell and tissue research.

[18] Fairman CL, Clagett-Dame M, Lennon VA, Epstein ML (1995). Appearance of neurons in the developing chick gut. Dev Dyn.

[19] Uesaka T, Nagashimada M, Enomoto H (2013). GDNF signaling levels control migration and neuronal differentiation of enteric ganglion precursors. J Neurosci.

[20] Anderson RB (2006). The cell adhesion molecule L1 is required for chain migration of neural crest cells in the developing mouse gut. Gastroenterology.

[21] Ovchinnikov D (2009). Alcian blue/alizarin red staining of cartilage and bone in mouse. Cold Spring Harbor protocols.

[22] Barlow AJ, Dixon J, Dixon MJ, Trainor PA (2012). Balancing neural crest cell intrinsic processes with those of the microenvironment in Tcof1 haploinsufficient mice enables complete enteric nervous system formation. Hum Mol Genet.

[23] Obermayr F, Hotta R, Enomoto H, Young HM (2013). Development and developmental disorders of the enteric nervous system. Nat Rev Gastroenterol Hepatol.

[24] Amiel J (2008). Hirschsprung disease, associated syndromes and genetics: a review. J Med Genet.

[25] McCallion AS, Stames E, Conlon RA, Chakravarti A (2003). Phenotype variation in two-locus mouse models of Hirschsprung disease: tissue-specific interaction between Ret and Ednrb. Proceedings of the National Academy of Sciences of the United States of America.

[26] Stewart AL, Young HM, Popoff M, Anderson RB (2007). Effects of pharmacological inhibition of small GTPases on axon extension and migration of enteric neural crest-derived cells. Developmental biology.

[27] Fu M (2010). Vitamin A facilitates enteric nervous system precursor migration by reducing Pten accumulation. Development (Cambridge, England).

[28] Young HM (2014). Colonizing while migrating: how do individual enteric neural crest cells behave?. BMC biology.

[29] Young HM, Jones BR, McKeown SJ (2002). The projections of early enteric neurons are influenced by the direction of neural crest cell migration. J Neurosci.

[30] Sasselli V (2013). Planar cell polarity genes control the connectivity of enteric neurons. The Journal of clinical investigation.

[31] Hatch J, Mukouyama YS (2015). Spatiotemporal mapping of vascularization and innervation in the fetal murine intestine. Dev Dyn.

[32] Durbec PL, Larsson-Blomberg LB, Schuchardt A, Costantini F, Pachnis V (1996). Common origin and developmental dependence on c-ret of subsets of enteric and sympathetic neuroblasts. Development (Cambridge, England).

[33] Young HM, Stamp LA, McKeown SJ (2016). ENS Development Research Since 1983: Great Strides but Many Remaining Challenges. Advances in experimental medicine and biology.

[34] Nagashimada M (2012). Autonomic neurocristopathy-associated mutations in PHOX2B dysregulate Sox10 expression. The Journal of clinical investigation.

[35] Dubreuil V (2008). A human mutation in Phox2b causes lack of CO2 chemosensitivity, fatal central apnea, and specific loss of parafacial neurons. Proceedings of the National Academy of Sciences of the United States of America.

[36] Onimaru H, Ikeda K, Kawakami K (2008). CO2-sensitive preinspiratory neurons of the parafacial respiratory group express Phox2b in the neonatal rat. J Neurosci.

[37] Gray PA, Rekling JC, Bocchiaro CM, Feldman JL (1999). Modulation of respiratory frequency by peptidergic input to rhythmogenic neurons in the preBotzinger complex. Science (New York, N.Y.

[38] Smith JC, Ellenberger HH, Ballanyi K, Richter DW, Feldman JL (1991). Pre-Botzinger complex: a brainstem region that may generate respiratory rhythm in mammals. Science (New York, N.Y.

[39] Brunjes PC (2012). The mouse olfactory peduncle. 2.The anterior limb of the anterior commissure. Frontiers in neuroanatomy.

[40] Ohnuma K, Imaizumi K, Masuno M, Nakamura M, Kuroki Y (1997). Magnetic resonance imaging abnormalities of the brain in Goldberg-Shprintzen syndrome (Hirschsprung disease, microcephaly, and iris coloboma). Am J Med Genet.

[41] Schuchardt A, D’Agati V, Larsson-Blomberg L, Costantini F, Pachnis V (1995). RET-deficient mice: an animal model for Hirschsprung’s disease and renal agenesis. Journal of internal medicine.

[42] Uesaka T, Nagashimada M, Yonemura S, Enomoto H (2008). Diminished Ret expression compromises neuronal survival in the colon and causes intestinal aganglionosis in mice. The Journal of clinical investigation.

[43] Ratcliffe EM (2006). Netrin/DCC-mediated attraction of vagal sensory axons to the fetal mouse gut. The Journal of comparative neurology.

[44] Goldberg D (2013). Slit/Robo-mediated chemorepulsion of vagal sensory axons in the fetal gut. Dev Dyn.

[45] Murphy MC, Fox EA (2010). Mice deficient in brain-derived neurotrophic factor have altered development of gastric vagal sensory innervation. The Journal of comparative neurology.

[46] Calmont A, Thapar N, Scambler PJ, Burns AJ (2011). Absence of the vagus nerve in the stomach of Tbx1−/− mutant mice. Neurogastroenterol Motil.

[47] Honma Y (2002). Artemin is a vascular-derived neurotropic factor for developing sympathetic neurons. Neuron.

[48] Glebova NO, Ginty DD (2004). Heterogeneous requirement of NGF for sympathetic target innervation *in vivo*. J Neurosci.

[49] Brunet I (2014). Netrin-1 controls sympathetic arterial innervation. The Journal of clinical investigation.

[50] Uesaka T, Young HM, Pachnis V, Enomoto H (2016). Development of the intrinsic and extrinsic innervation of the gut. Developmental biology.

[51] Dyachuk V (2014). Neurodevelopment. Parasympathetic neurons originate from nerve-associated peripheral glial progenitors. Science (New York, N.Y.

[52] Espinosa-Medina I (2014). Neurodevelopment. Parasympathetic ganglia derive from Schwann cell precursors. Science (New York, N.Y.

[53] Espinosa-Medina I (2017). Dual origin of enteric neurons in vagal Schwann cell precursors and the sympathetic neural crest. Proceedings of the National Academy of Sciences of the United States of America.

[54] Uesaka T, Nagashimada M, Enomoto H (2015). Neuronal Differentiation in Schwann Cell Lineage Underlies Postnatal Neurogenesis in the Enteric Nervous System. J Neurosci.

[55] Furlan, A. *et al*. Multipotent peripheral glial cells generate neuroendocrine cells of the adrenal medulla. *Science (New York, N.Y* **357** (2017). 10.1126/science.aal3753 PMC6013038 28684471

[56] Hirokawa N, Noda Y (2008). Intracellular transport and kinesin superfamily proteins, KIFs: structure, function, and dynamics. Physiological reviews.

[57] Kubin L, Alheid GF, Zuperku EJ, McCrimmon DR (2006). Central pathways of pulmonary and lower airway vagal afferents. J Appl Physiol (1985).

[58] Dutschmann M, Morschel M, Rybak IA, Dick TE (2009). Learning to breathe: control of the inspiratory-expiratory phase transition shifts from sensory- to central-dominated during postnatal development in rats. The Journal of physiology.

[59] Wong KA (1998). Pulmonary vagal innervation is required to establish adequate alveolar ventilation in the newborn lamb. J Appl Physiol (1985).

[60] Rao M, Gershon MD (2016). The bowel and beyond: the enteric nervous system in neurological disorders. Nat Rev Gastroenterol Hepatol.

