# Publisher Correction: Kif1bp loss in mice leads to defects in the peripheral and central nervous system and perinatal death

**DOI:** 10.1038/s41598-018-26661-5

**Published:** 2018-06-08

**Authors:** Caroline S. Hirst, Lincon A. Stamp, Annette J. Bergner, Marlene M. Hao, Mai X. Tran, Jan M. Morgan, Matthias Dutschmann, Andrew M. Allen, George Paxinos, Teri M. Furlong, Sonja J. McKeown, Heather M. Young

**Affiliations:** 1 Department of Anatomy and Neuroscience, The University of Melbourne, Victoria, 3010 Australia; 2 Florey Institute of Neuroscience and Mental Health, The University of Melbourne, Victoria, 3010 Australia; 3 Department of Physiology, The University of Melbourne, Victoria, 3010 Australia; 4 Neuroscience Research Australia and School of Medical Sciences, The University of New South Wales, 2031 NSW, Australia; 5 Cancer Program, Monash Biomedicine Discovery Institute and Department of Anatomy and Developmental Biology, Monash University, Victoria, 3800 Australia

Correction to: *Scientific Reports* 10.1038/s41598-017-16965-3, published online 30 November 2017

The original PDF version of this Article contained a truncated Figure 2. This error has now been corrected in the PDF version of the Article; the HTML version was correct from the time of publication.

