# Supplementary material for: Kif1bp loss in mice leads to defects in the peripheral and central nervous system and perinatal death
Source: Sci Rep. 2017 Nov 30;7:16676. doi: 10.1038/s41598-017-16965-3 (PMC5709403; doi:10.1038/s41598-017-16965-3)
Supplement: Supplementary file 1 — Supplementary information [file 41598_2017_16965_MOESM1_ESM.pdf]

SUPPLEMENTARY INFORMATION

Kif1bp loss in mice leads to defects in the peripheral and central nervous system and perinatal death

Caroline S. Hirst[1*], Lincon A. Stamp[1*], Annette J. Bergner[1], Marlene M. Hao[1], Mai X. Tran[1], Jan M. Morgan[1], Matthias Dutschmann[2], Andrew M. Allen[3], George Paxinos[4], Teri M. Furlong[4], Sonja J. McKeown[1,5§], Heather M. Young[1§]

1. Department of Anatomy and Neuroscience, The University of Melbourne, Victoria, 3010, Australia
2. Florey Institute of Neuroscience and Mental Health, The University of Melbourne, Victoria, 3010, Australia
3. Department of Physiology, The University of Melbourne, Victoria, 3010, Australia
4. Neuroscience Research Australia and School of Medical Sciences, The University of New South Wales, 2031, NSW, Australia
5. Cancer Program, Monash Biomedicine Discovery Institute and Department of Anatomy and Developmental Biology, Monash University, Victoria, 3800, Australia

* Equal first authors

[§] Equal corresponding authors:

h.young@unimelb.edu.au; telephone +613 8344 0007

sonja.mckeown@monash.edu; telephone +613 9905 0202

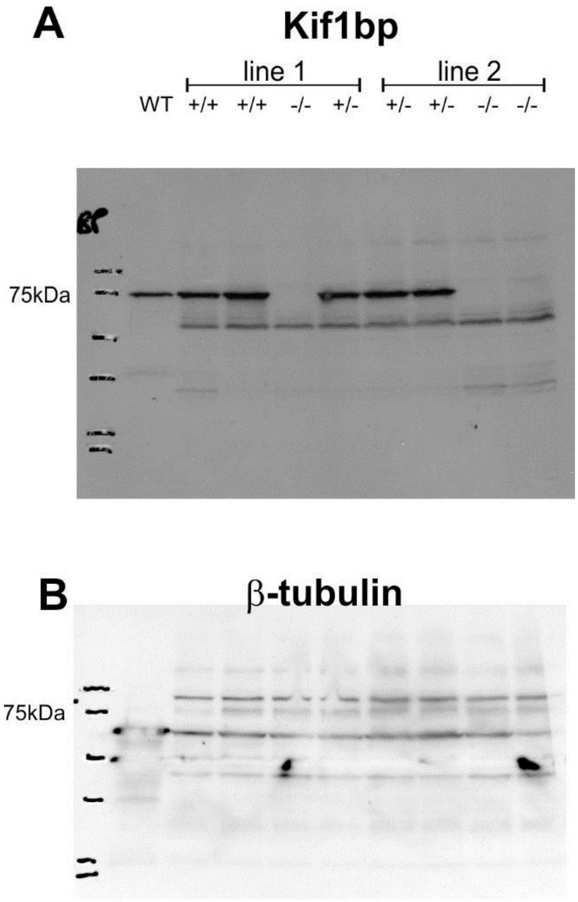

Supplementary figure 1. Full length gels of Western blots using antibodies directed against mKif1bp (A) and b-tubulin (B). b-tubulin was used as a loading control. There is an absence of Kif1bp protein (74kDa band) in null mutant mice from each of the two mouse lines. An additional, smaller band was observed in all of the embryonic samples, but not the adult WT liver.

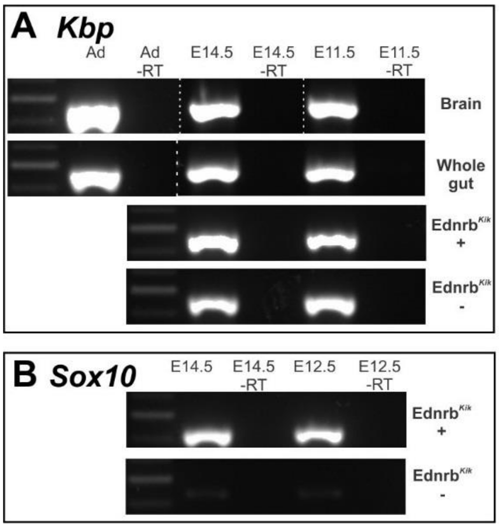

Supplementary figure 2. Expression of *Kbp* using RT-PCR. **A, top 2 rows.** RT-PCR demonstration of *Kbp* expression by the adult brain, and E11.5 and E14.5 whole gut from C57BL/6 mice. **A, bottom 2 rows.** To determine if *Kbp* is expressed by ENCCs, *Ednrb-hKikGR* mice were used, in which the fluorescent protein, Kikume, is exclusively expressed by ENCCs. RT-PCR of FACS-sorted Kik+(ENCCs) and Kik- (non-ENCC) cells from the E12.5 and E14.5 gut revealed *Kbp* expression by both fractions. Thus, *Kbp* is expressed by both ENCC and non-ENCCs in the embryonic gut. **B.** In contrast to *Kbp*, expression of the neural crest gene, *Sox10*, is barely detectable in the non-ENCC fraction. For each tissue and primer set, negative controls were performed in the absence of reverse transcripase (-RT). Please see page 4 of Supplementary information for Methods.

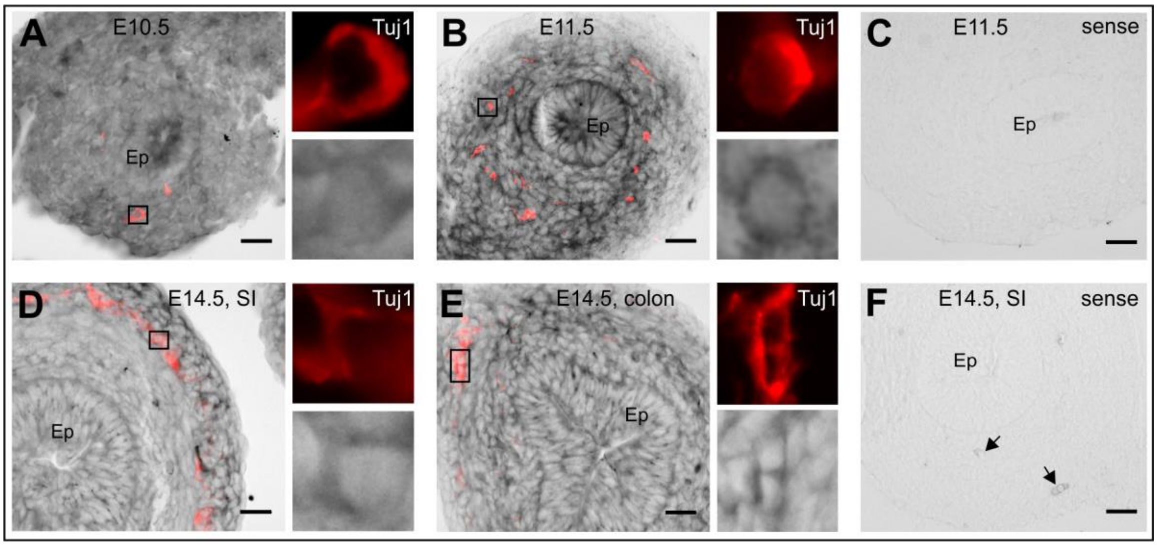

Supplementary figure 3. Expression of *Kbp* by the fetal gut using in situ hybridization. Guts were hybridized with a *Kbp* cRNA probe as wholemount preparations and then sectioned and processed for fluorescence immunohistochemistry using an antibody to the pan neuronal marker, Tuj1. **A.** At E10.5, *Kbp* was expressed by most mesenchymal cells and the epithelium (*Ep*). The expression of *Kbp* by Tuj1+ cells (inset) was similar to, or only slightly higher than, the expression of *Kbp* by neighbouring, Tuj1-, cells. **B, D, E**. In the E11.5 small intestine (**B**) and E14.5 small intestine (**D**) and colon (**E**), the expression of *Kbp* by Tuj1+ cells was higher than by Tuj1- cells. Furthermore, expression of *Kbp* was higher in the outer mesenchyme than the inner mesenchyme of the E14.5 small intestine. **C, F.** Wholemount preparations hybridized in parallel using sense probes were used as negative controls. The only staining in tissue processed using the sense probe was non-specific staining of blood cells (**F**, *arrows*). Immunostaining using the neural crest markers, Sox10 and p75, was not successful on tissue that had been hybridized so it was not possible to assess *Kbp* expression by non-neuronal ENCCs. Scale bars (apply to large panels only): 20 μm. Please see pages 4-5 of Supplementary information for Methods.

Supplementary Methods

RNA extraction and cDNA synthesis

Tissue from adult, E11.5 or E14.5 C57BL/6 mice was dissected quickly in sterile DMEM/F12 (Invitrogen) and stored at -20°C in RNAlater (Qiagen). Tissue selected for isolation included adult brain and duodenum (myenteric plexus with attached smooth muscle layers), E11.5 and E14.5 whole intestines (entire intestine caudal to the stomach), brain (E11.5 entire head), liver, heart and yolk sac. In addition, approximately $1x10^6$ *Ednrb*^{Kik} positive and negative cell populations were FACS sorted from freshly dissociated E12.5 and E14.5 *Ednrb-hKikGR* mice and immediately frozen at -80°C.

Total RNA was extracted using QIAshredder and RNeasy Mini Kit (Qiagen), including the on-column DNase treatment, according to manufacturer's instructions. The concentration of total RNA in each sample was measured using a NanoDrop ND-1000 spectrophotometer. RNA concentration and integrity were measured using a 2200 TapeStation Instrument (Agilent), when used for ddPCR anyalsis. cDNA was synthesised using the iScript Advanced cDNA Synthesis Kit for RT-qPCR (Bio-Rad); 500ng or 100ng (cells) of total RNA was used in a final reaction volume of 20 µl according to the manufacturer's instructions. Control reactions using no reverse transcriptase (-RT) were run in parallel for each tissue.

Reverse transcription-polymerase chain reaction (RT-PCR)

RT-PCR was conducted using intron-spanning specific primer pairs (Supplementary Table 1, KBP and Sox10) and a touchdown PCR (TD-PCR) cycling program (Supplementary Table 2). A standard RT-PCR protocol was used consisting of cDNA (1-2 ul), MangoTaq™ (0.2 ul, Bioline), dNTP Mix (0.8 mM, Bioline), $MgCl_2$ (2 mM, Bioline), and primer pairs (1 µM each) in a final reaction volume of 20 µl. RT-PCR products (5µl) were resolved by gel electrophoresis on a 1% agrose gel, containing GelRed™ (0.5x, Biotium), together with Ready-to-Use 100 bp DNA Ladder (Biotium) to estimate RT-PCR product size. Control –RT reactions were run in parallel for each RT-PCR reaction.

In situ hybdrization (ISH)

To generate a *Kbp* probe for ISH, primer pairs were designed with T3 (fwd) or T7 (rev) sequences attached to the 5' end of the primers (see KBP_2 primer set, Supplementary Table 1). The primers were used to amplify *Kbp* from adult brain cDNA using the methods described above. The resulting PCR products were sequenced to confirm specificity for *Kbp*. A plasmid containing the mouse *Sox10* gene was also used. Digoxigenin-labeled single-stranded riboprobes were prepared using a digoxigenin RNA labelling kit (Roche) according to manufacturer's instructions. After labeling, riboprobes were purified on Chroma spin-100 columns (BD, Bioscience).

For wholemount ISH, embryos were washed twice in DEPC-treated phosphate buffered saline (PBS), fixed overnight in 4% paraformaldehyde, washed twice in DEPC-PBT (PBS, 0.1% Tween-20), dehydrated in methanol, and then stored at −20°C. Embryos were re-hydrated to PBT, bleached in 6% hydrogen peroxide, treated with 10 µg of proteinase K, re-fixed, and hybridized with the digoxigenin-labeled riboprobe overnight at 70°C. Embryos were washed thoroughly at high stringency at 70°C/65°C before incubation with the anti-digoxigenin

antibody (Roche) overnight at 4°C. To reveal sites of expression, embryos were treated with INT/BCIP solution (Roche) for several days (method adapted from {Wilkinson, 1992 #5356}). Selected embryos were photographed with Leica MZ FLIII stereomicroscope and Leica DC 500 camera and embedded in a gelatin–sucrose–albumin (1:1.5:60) solution, solidified by addition of 2.5% of glutaraldehyde. A Vibratome was used to obtain 50-µm thickness sections, which were mounted with 50% glycerol, and photographed with an Axiophot (Zeiss) photomicroscope. Sense-strand riboprobes gave no specific signal.

Supplementary Table 1. Sequences of primers for RT-PCR and in situ hybridisation. Underlined regions indicate T3 or T7 sequences.

Gene	Forward Primer Sequence (5' – 3')	Reverse Primer Sequence (5' – 3')	Product Size (bp)
KBP	AATTAACCCTCACTAAAGGGAGAGTTCGAGAAGGCCGCTCATT	TAATACGACTCACTATAGGGAGAACTGCACAGCCCTTTTCCTG	425
Sox10	CATGGCCGAGGAACAAGACC	CGTTCAGCAACCTCCAGAGC	440
KBP_2	AATTAACCCTCACTAAAGGGAGAAGATGGTCAGTCTCCTCCCA	TAATACGACTCACTATAGGGAGAATTCAACACAGCAACAGGCTC	433

Supplementary Table 2. TD-PCR cycling program parameters for RT-PCR

Phase 1	Step	Temperature	Time
1	Denature	95°C	5 min
2	Denature	95°C	30 s
3	Anneal	65°C	45 s
4	Elongate	72°C	30 s
Repeat 2-4 (9 times) reducing annealing temperatures by 1°C/cycle			
Phase 2	Step	Temperature	Time
5	Denature	95°C	30 s
6	Anneal	55°C	45 s
7	Elongate	72°C	30 s
Repeat steps 5-6 either 24 times (TDa) or 29 times (TDb)			
Termination	Step	Temperature	Time
8	Elongate	72°C	10 min
9	Halt reaction	4°C	10 min
